# Lithium Treatment of APPSwDI/NOS2−/− Mice Leads to Reduced Hyperphosphorylated Tau, Increased Amyloid Deposition and Altered Inflammatory Phenotype

**DOI:** 10.1371/journal.pone.0031993

**Published:** 2012-02-09

**Authors:** Tiffany L. Sudduth, Joan G. Wilson, Angela Everhart, Carol A. Colton, Donna M. Wilcock

**Affiliations:** 1 University of Kentucky Sanders-Brown Center on Aging, Department of Physiology, Lexington, Kentucky, United States of America; 2 Duke University Medical Center, Department of Medicine, Division of Neurology, Durham, North Carolina, United States of America; Emory University, United States of America

## Abstract

Lithium is an anti-psychotic that has been shown to prevent the hyperphosphorylation of tau protein through the inhibition of glycogen-synthase kinase 3-beta (GSK3β). We recently developed a mouse model that progresses from amyloid pathology to tau pathology and neurodegeneration due to the genetic deletion of NOS2 in an APP transgenic mouse; the APPSwDI/NOS2−/− mouse. Because this mouse develops tau pathology, amyloid pathology and neuronal loss we were interested in the effect anti-tau therapy would have on amyloid pathology, learning and memory. We administered lithium in the diets of APPSwDI/NOS2−/− mice for a period of eight months, followed by water maze testing at 12 months of age, immediately prior to sacrifice. We found that lithium significantly lowered hyperphosphorylated tau levels as measured by Western blot and immunocytochemistry. However, we found no apparent neuroprotection, no effect on spatial memory deficits and an increase in histological amyloid deposition. Aβ levels measured biochemically were unaltered. We also found that lithium significantly altered the neuroinflammatory phenotype of the brain, resulting in enhanced alternative inflammatory response while concurrently lowering the classical inflammatory response. Our data suggest that lithium may be beneficial for the treatment of tauopathies but may not be beneficial for the treatment of Alzheimer's disease.

## Introduction

Alzheimer's disease (AD) is a progressive, neurodegenerative disorder characterized clinically by an advancing cognitive decline. Pathologically, AD is identified by the presence of extracellular amyloid plaques composed of aggregated Aß peptides and intracellular neurofibrillary tangles (NFTs) composed of hyperphosphorylated, aggregated tau protein [1]. Both amyloid plaques and neurofibrillary tangles are the targets of disease-modifying therapy development for the treatment of AD.

Tau protein is a microtubule associated protein that is involved in the stabilization of microtubules in a phosphorylation dependent manner [2]. Abnormal and/or excessive phosphorylation of tau protein leads to its permanent dissociation from the microtubule, redistribution to the cell soma and aggregation leading to the formation of an insoluble NFT. While NFTs are pathological hallmarks of AD, they also characterize other tauopathies such as progressive supranuclear palsy (PSP) and frontotemporal dementia (FTD) [3]. The abnormal phosphorylation of tau is thought to result from disruption of the kinase-phosphatase systems. Therefore, inhibition of kinases has been one target for the reduction of hyperphosphorylated tau, and therefore NFTs [4].

Lithium is an antipsychotic medication that has been shown to inhibit glycogen-synthase kinase 3-beta (GSK3β); a key kinase for the phosphorylation of tau [5]. We recently developed a mouse model for the study of AD that develops tau pathology in addition to amyloid pathology [6]. This mouse develops extensive amyloid deposition, hyperphosphorylated tau and neuronal loss by 12 months of age. We have previously shown that targeting the amyloid pathology using anti-Aβ immunotherapy results in amelioration of not only the amyloid pathology but also the tau pathology, neuronal loss and improvement in learning and memory [7]. Our goal in the current study was to determine what impact targeting the tau pathology using lithium would have on the amyloid pathology, neuronal loss, learning and memory. Our findings replicate those of Noble et al who showed reductions in hyperphosphorylated tau in response to lithium treatment in mice expressing the P301L human tau mutation [8]. However, we found that lithium had no impact on Aβ levels and, in fact, increased the density of amyloid deposits. We believe that this may be due to an altered inflammatory state of the brain resulting from the lithium treatment; an effect separate from the action at the GSK3β site.

## Results

**Figure 1:**
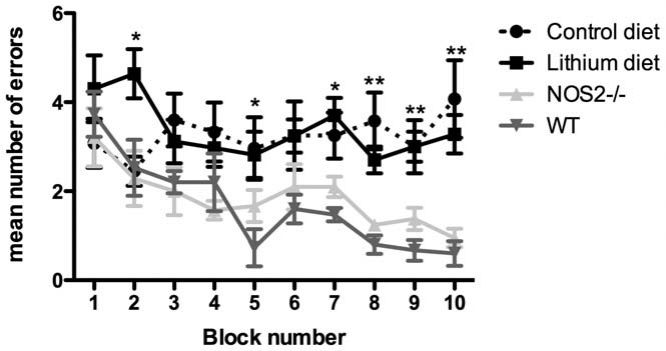
Lithium does not improve outcomes on the radial-arm water maze spatial memory task. APPSwDI/NOS2−/− mice receiving either control diet (N = 8, 4 male and 4 female) or lithium supplemented diet (N = 11, 4 female and 7 male), NOS2−/− (N = 7, 3 female and 4 male) and wildtype mice (N = 7, 3 female and 4 male) aged 12 months were tested on the two-day radial-arm water maze task. Data are shown as mean number of errors per block number. Each block comprises three trials. * indicates P<0.05, ** indicates P<0.01 for both the APPSwDI/NOS2−/− treatment groups when compared to NOS2−/− and wildtype mice for each block number.

Radial-arm water maze testing showed that non-transgenic, wildtype (WT) and NOS2−/− mice aged 12 months learned the task as is evidenced by the decline in number of errors over the two days of testing, ending with a mean of less than 1 error at the end of day 2 (figure 1). Importantly, all mice, transgenic and non-transgenic begin testing with no statistical significant difference. APPSwDI/NOS2−/− mice aged 12 months were significantly impaired in the radial-arm water maze as previously shown ([6] figure 1). As can be seen in figure 1, treatment of APPSwDI/NOS2−/− mice with lithium also resulted in impaired memory and learning when compared to either the non-transgenic or NOS2−/− mice but the number of errors was not statistically different from untreated APPSwDI/NOS2−/− mice. Thus, treatment of mice with lithium did not result in significant changes in memory as measured by the radial-arm water maze.

**Figure 2:**
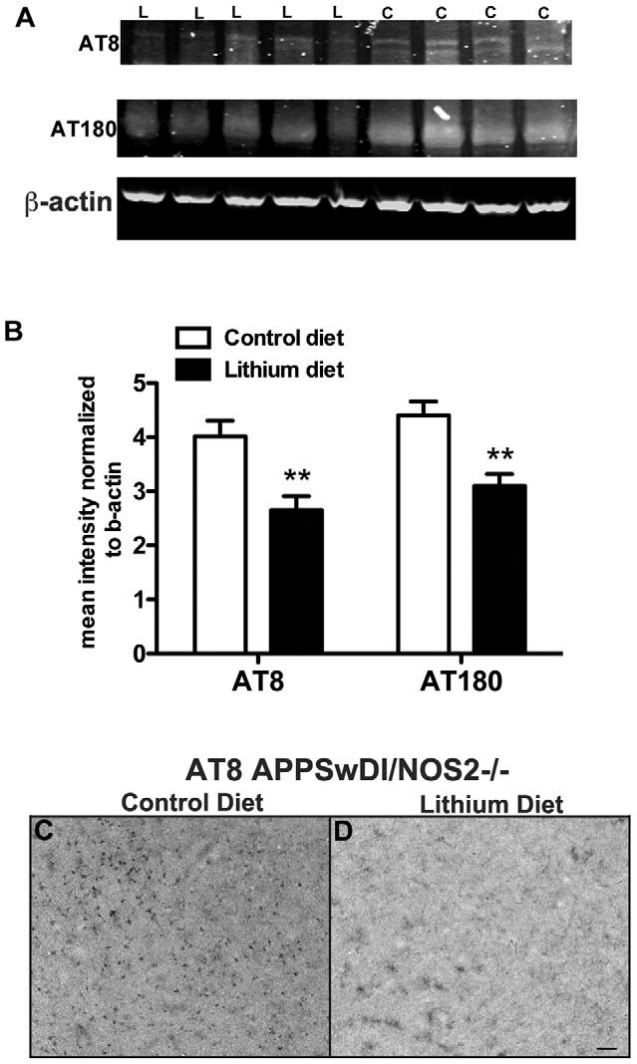
Lithium treatment reduces the levels of abnormally phosphorylated tau protein in APPSwDI/NOS2−/− mice. Panel A shows representative Western blot images for AT8, AT180 and ß-actin for APPSwDI/NOS2−/− mice receiving either lithium diet (L) or control diet (C). Panel B shows the relative quantification of the band intensity for AT8 and AT180 normalized to the ß-actin band. N = 11 lithium treated mice, N = 8 control treated mice. ** indicates P<0.01 when compared to control treated mice. Panels C and D show immunocytochemistry for AT8 in the frontal cortex region of APPSwDI/NOS2-/mice receiving control diet (C) or lithium supplemented diet (D) for 8 months. Scale bar in D for C and D = 50 µm.

Since lithium has been associated with reduced tau hyperphosphorylation and subsequent aggregation we assessed tau phosphorylation by semi-quantitative Western blot. We observed a reduced signal for both AT8 (which recognizes tau phosphorylation at Ser202/Thr205) and AT180 (which recognizes tau phosphorylation at Thr231) (figure 2A). Both sites are associated with pathological phosphorylation in AD and other tauopathies. Densitometric quantification of the band intensity when normalized to ß-actin showed an approximate 35% reduction in tau hyperphosphrylation for both AT8 and AT180 (figure 2B). Immunohistochemistry for AT8 revealed a noticeable reduction in immunopositive neurons in the cortical regions of the brain. A representative example of stain intensity in shown in Figure 2 for untreated (2C_) vs lithium treated mice (2D) mice.

**Figure 3:**
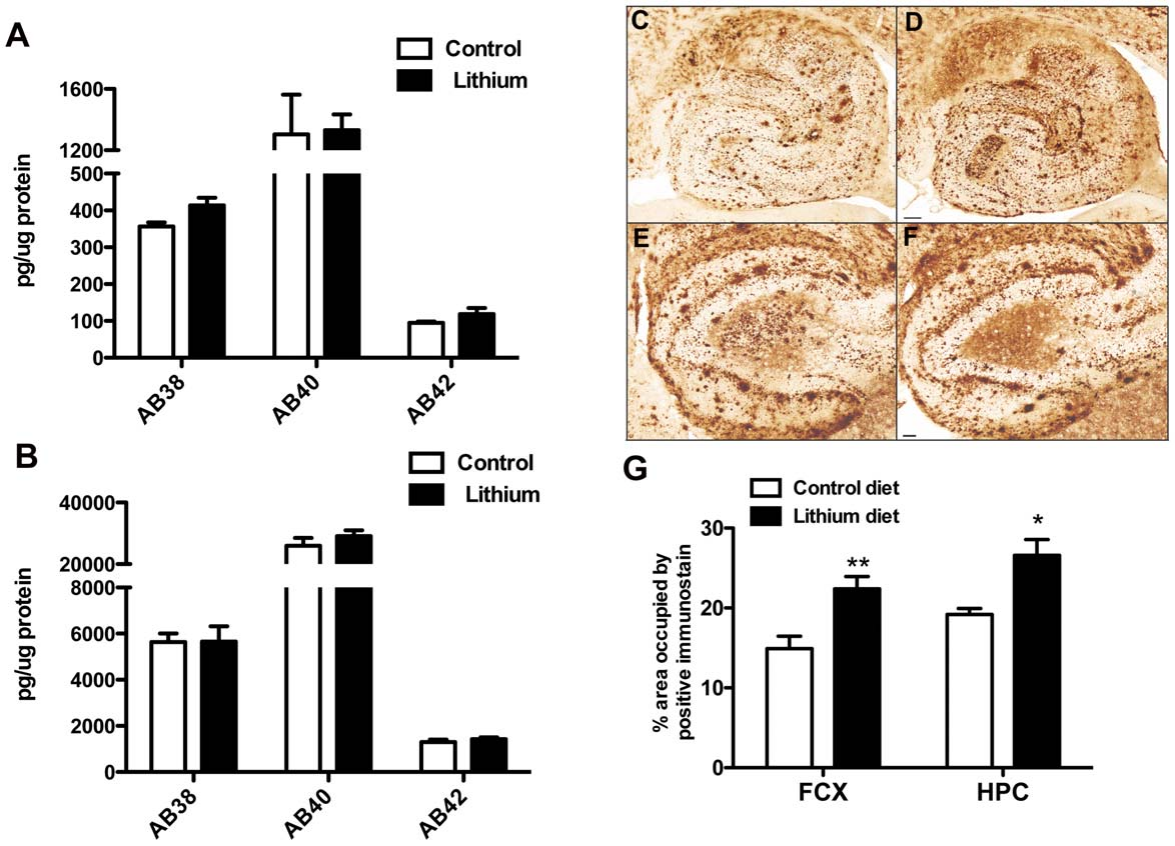
Lithium treatment of APPSwDI/NOS2−/− mice does not change Aβ levels but does increase Aβ deposition. Panels A and B show biochemical ELISA measurement of Aβ38, 40 and 42 in soluble (A) and insoluble (B) protein extracts. Panels C–F show representative images of the whole hippocampus at 40× magnification (C and D) and the dentate gyrus of the hippocampus at 100× magnification (E and F) of APPSwDI/NOS2−/− mice receiving control diet (C and E) or lithium diet (D and F). Scale bar in D for C and D = 120 µm, scale bar in F for E and F = 50 µm. Panel G shows quantification of percent area occupied by positive stain for mice receiving control diet (N = 8) or lithium diet (N = 11) for 8 months. * indicates P<0.05, ** indicates P<0.01 when compared to APPSwDI/NOS2−/− mice receiving control diet.

To determine the effects of lithium on Aβ, we assessed Aβ by both biochemical and immunohistochemical methods. As can be seen in figures 3A and B we did not observe significant differences in soluble or insoluble Aβ38, Aβ40 or Aβ42 resulting from the lithium treatment. We did, however, observe a significant increase in the amount of Aβ immunohistochemical staining. This was evident throughout the brain but most noticeable in the hippocampus (figure 3C and D), especially the dentate gyrus region (figure 3E and F). When we quantified the percent area occupied by immunoreactive product in both the frontal cortex and hippocampus we found a statistically significant 30% increase in both regions (figure 3G).

**Figure 4:**
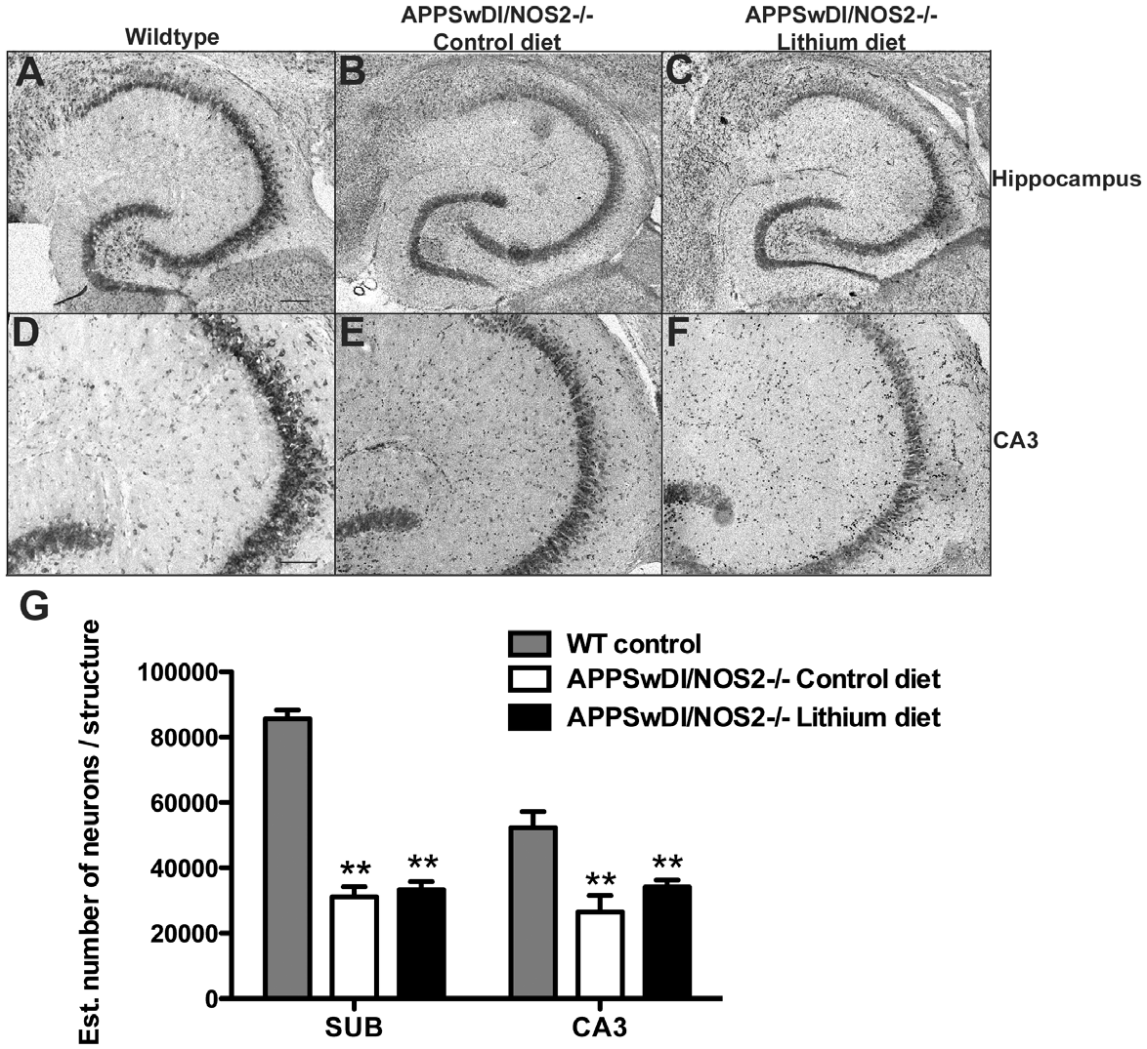
Lithium treatment does not alter neuronal loss in the APPSwDI/NOS2−/− mice. Panels A–F show Nissl staining for wildtype (A and D), APPSwDI/NOS2−/− mice receiving control diet (B and E) and APPSwDI/NOS2−/− mice receiving lithium diet (C and F). Panels A–C show the whole hippocampus at 40× magnification (scale bar in A for A–C = 120 µm). Panels D–F show the CA3 region of the hippocampus at 100× magnification (scale bar in D for D–F = 50 µm). Panel G shows stereological counts of neuron number in the subiculum and CA3 of wildtype mice and APPSwDI/NOS2−/− mice receiving either control diet or lithium diet. ** indicates P<0.01 compared to wildtype mice.

We have previously shown that the APPSwDI/NOS2−/− mouse develops significant neuronal loss by 12 months of age [6]. We have also shown that anti-Aβ immunotherapy ameliorates this neuronal loss. We performed Nissl staining on serial sections and performed stereological neuron counts on the CA3 region of the hippocampus and the subiculum; the two regions previously found to show the most dramatic neuronal loss in the APPSwDI/NOS2−/− mice [6]. We found that the APPSwDI/NOS2−/− mice receiving control diet showed a significant neuronal loss comparable to that previously reported in 12 month old mice (figure 4B, E and G). Interestingly, we observed the same degree of neuronal loss in APPSwDI/NOS2−/− mice receiving the lithium supplemented diet, indicating that lithium treatment did not prevent neuronal loss in the APPSwDI/NOS2−/− mice (figure 4C, F and G).

**Figure 5:**
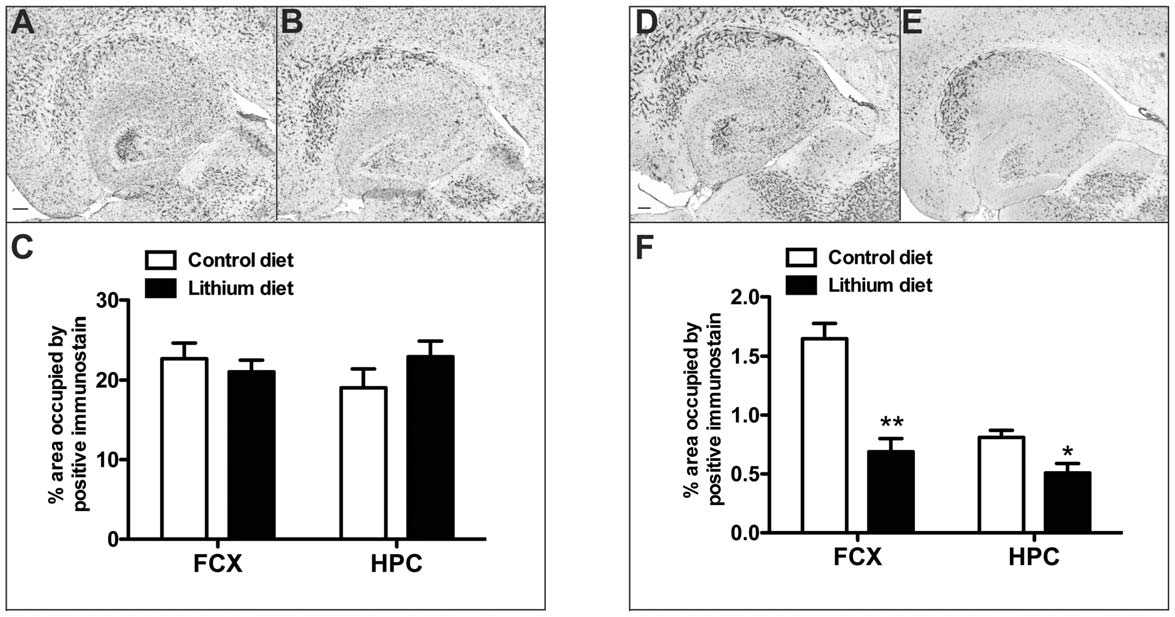
Microglial activation is reduced following lithium treatment. Panels A and B show CD11b immunocytochemistry in the hippocampus of APPSwDI/NOS2−/− mice receiving either control diet (A) or lithium diet (B) for 8 months. Scale bar in panel A for A and B = 120 µm. Panel C shows quantification of the percent area occupied by positive stain for CD11b in the frontal cortex and hippocampus. Panels D and E show CD45 immunocytochemistry in the hippocampus of APPSwDI/NOS2−/− mice receiving either control diet (D) or lithium diet (E) for 8 months. Scale bar in panel D for D and E = 120 µm. Panel F shows quantification of the percent area occupied by positive stain for CD45 in the frontal cortex and hippocampus. * indicates P<0.05 and ** indicates P<0.01 when compared to APSPwDI/NOS2−/− mice receiving control diet.

To determine whether lithium had an influence on the microglial population we performed immunocytochemistry for CD11b and CD45. CD11b is a standard marker for all states of microglial cells that increases in intensity with activation [9]. CD45 is typically not expressed by resting microglia in mouse brain but is expressed upon activation [9]. We observed CD11b staining throughout the brain, with increased intensity in the subiculum and dentate gyrus of APPSwDI/NOS2−/− mice, which corresponds to the regions of the most intense amyloid deposition (figure 5A). Lithium treatment did not alter the pattern or observed intensity of the CD11b staining (figure 5B). Quantification of percent area occupied by positive immunostain showed no significant difference between APPSwDI/NOS2−/− mice receiving either control or lithium supplemented diet (figure 5C). In contrast to CD11b, we found that lithium treatment decreased the amount of CD45 immunostaining in the brain and the intensity of the staining (figure 5D and E). Quantification of the percent area occupied by positive immunostain showed a significant decrease in the amount of CD45 staining in both the frontal cortex and hippocampus (figure 5F).

**Figure 6:**
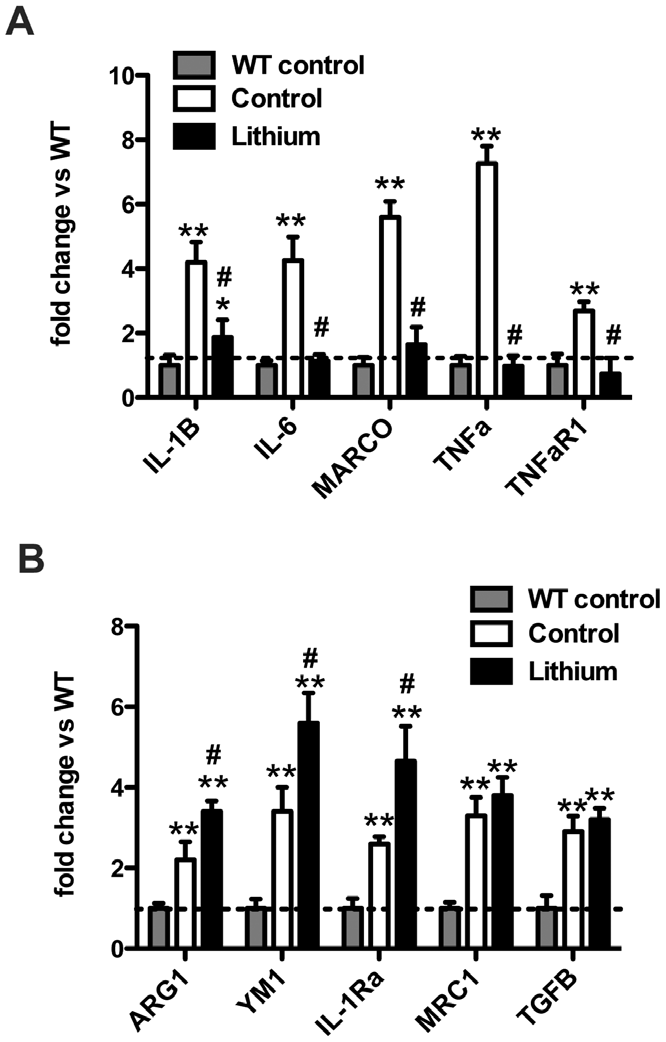
Lithium significantly alters the inflammatory state of the APPSwDI/NOS2−/− mouse brain. Panels A and B show relative gene expression changes of classical inflammatory genes (A) and alternative inflammatory genes (B) in wildtype mice and APPSwDI/NOS2−/− mice receiving either control or lithium diet. Data are shown as fold change compared to the mean of the wildtype mice. ** indicates P<0.01 when compared to wildtype expression. # indicates P<0.05 when compared to APPSwDI/NOS2−/− mice receiving control diet.

To further characterize the inflammatory changes that might occur as a result of the lithium treatment we performed quantitative real-time RT-PCR for genes associated with classical and alternative inflammatory responses. We have previously shown that transgenic mice show diverse inflammatory responses and anti-Aβ immunotherapy significantly alters these responses [10]. We found that untreated APPSwDI/NOS2−/− mice show significant increases in mRNA for classical inflammatory genes interleukin 1β (IL-1β), interleukin 6 (IL-6), macrophage receptor with collagenous structure (MARCO), tumor necrosis factor α (TNFα) and TNFα receptor 1 (TNFaR1) (figure 6A). Interestingly, lithium treatment significantly reduced the expression of each of these genes to levels observed in wildtype mice (figure 6A). While lithium treatment significantly lowered classical inflammatory gene expression, it increased alternative gene expression. We found that APPSwDI/NOS2−/− mice on a normal diet showed significantly elevated expression of alternative activation genes arginase 1 (ARG1), YM1, IL-1Ra (the IL-1 receptor antagonist), mannose receptor C1 (MRC1) and transforming growth factor-β1 (TGFβ1) compared to wildtype mice (figure 6B) Interestingly, in contrast to the effect lithium had on classical genes, we found that lithium further increased the mRNA expression of some alternative inflammation genes in APPSwDI/NOS2−/− mice (figure 6B). With respect to ARG1, YM1 and IL-1Ra there was a statistically significant increase in lithium treated mice compared to untreated APPSwDI/NOS2−/− mice (figure 6B).

## Discussion

The effect of lithium to significantly lower hyperphosphorylation of tau protein in mouse models of tauopathies was clearly shown by Noble et al [8] who linked the effect of lithium on tau to its action as an inhibitor of GSK3β [5]. More recently, it was also shown that lithium increased Hsp70 [11] Hsp70 has been shown to be important in the ubiquitination and subsequent degradation of tau protein [12], [13]. The published data on the beneficial effects of lithium on tau pathology have resulted in the performance of several clinical studies of lithium as a potential treatment for Alzheimer's disease [14], [15].

We recently developed a mouse model of Alzheimer's disease that develops amyloid pathology as well as endogenous mouse tau pathology and significant neurodegeneration [6]. Treatment of the APPSwDI/NOS2−/− mouse model with anti-Aβ therapies was subsequently shown to lower not only amyloid pathology but also tau pathology as well as to reduce neuronal loss [7]. We were particularly interested to determine whether a therapy targeting tau would have similar effects on amyloid pathology and neurodegeneration so we elected to treat our mice with lithium supplemented diet. Mice were treated for 8 months with either lithium supplemented, or control diet beginning at 4 months of age. While our data show a reduction in hyperphosphorylated tau levels on lithium treated APPSwDI/NOS2−/− mice, spatial memory of the mice remained significantly impaired. In addition, neurodegeneration was not blocked by lithium and amyloid deposition was increased. These data are similar to studies on the 3XTg transgenic mouse model that expresses mutated human APP, PS1 and mutated human tau where lithium treatment was also associated with reduced tau pathology but no change in either Aβ levels or memory [16]. Our current study expands on the findings of Caccamo et al by showing that lithium also influences neuroinflammation. Treatment of APPSwDI/NOS2−/− mice with lithium altered the inflammatory state of the brain by reducing CD45 positive microglia, lowering classical inflammatory markers and increasing alternative inflammatory markers.

GSK3β is a serine/threonine protein kinase that has been shown to be involved in the phosphorylation of tau protein. In particular, GSK3β has been linked to the abnormal phosphorylation of tau in AD, and other tauopathies [17], [18]. While lithium has been shown to prevent the hyperphosphorylation of tau in mouse models expressing mutant human tau, we report here that lithium also significantly reduces abnormal tau phosphorylation in a mouse model that develops tau pathology in normal, native mouse tau. This is an important finding since mutations of human tau are linked to tauopathies and have not yet been shown to occur in AD. We believe that the beneficial effect of lithium on the abnormal phosphorylation of normal tau as well as mutant tau further reinforces this approach for the treatment of tauopathies.

Despite these beneficial effects of lithium treatment on tau, lithium did not alter total brain levels of soluble and insoluble Aβ38, 40 and 42 and did not protect the neurons from degeneration. Thus, the tau-lowering action of lithium appears to be independent of Abeta and is unlikely to have altered the production or clearance kinetics of the Aβ peptides. Interestingly, the effect of lithium to increase observable amyloid deposition in the brain suggests that lithium may directly or indirectly alter the environment of the brain to promote the deposition of Aβ.

Neuroinflammation is known to be present in the AD brain and has been hypothesized to be intimately involved in the control of AD pathologies [19]. There are numerous studies reporting conflicting effects of inflammation on amyloid pathology. Lipopolysaccharide (LPS) has been shown to both reduce [20], [21] and increase [22] amyloid deposition in APP transgenic mice. We have previously shown that anti-Aβ immunotherapy increases the pro-inflammatory phenotype of microglia, which appears to be at least partially responsible for the reductions in amyloid deposition [23], [24]. In the human clinical trials for immunotherapy there is also evidence that microglia are involved in the clearance of amyloid deposits [25]. More recently, we have shown that immunotherapy switches the inflammatory state of the brain away from an alternative inflammatory state while concurrently driving a classical inflammation [10]. In contrast to immunotherapy, we show here that lithium significantly increased gene markers characteristic of alternative activation and acquired deactivation immune states, both of which are associated with immunosuppression [26]. The concomitant loss of classical activation gene markers, further strengthens a functional phenotypic change away from removal of Abeta towards one of amyloid deposition [10]. Although the exact functional role of CD45 expression has not been well defined, the loss of CD45 immunoreactivity may also signal a switch to an immunosuppressive state.

Lithium is known to influence inflammation. Rapaport and Manji showed that lithium results in an increase in Th2 cytokines IL-4 and IL-10 along with a decrease in the Th1 cytokines IFNγ and IL-2 in an ex vivo assay on whole blood cultures [27]. Further supporting the effect of lithium on neuroinflammation is the recent finding that lithium reduced microglial activation and inhibited the production of classical inflammatory cytokines IL-1β and MCP-1 in a rat model of hypoxia-ischemia [28]. Our data shows similar effects, where classical inflammatory marker gene expression is significantly reduced following lithium treatment and alternative inflammatory marker gene expression is significantly increased. Since IL-4 and IL-10 are critical mediators of the alternative inflammatory response we can conclude that the findings of Rapaport and Manji expand to the brain, where Th1 cytokines are reduced and Th2 cytokines are elevated by lithium. Alternative inflammatory genes are often associated with wound repair and matrix remodeling. In fact, arginase 1 has been associated with the onset and progression of fibrosis in cystic fibrosis [29] and schistosoma infection [30]. YM1, also known as chitinase-3-like-3 (Chi3l3) is associated with matrix remodeling during parasitic infections [31] and in the development of dermatitis [32]. We hypothesize that the pro-fibrotic properties of the alternative inflammatory mediators promotes the fibrillogenesis of Aβ in the brain resulting in increased deposition of Aβ. The exacerbation of alternative inflammation in the presence of reduced classical inflammation by lithium treatment in the current study would support this hypothesis.

In summary, we find that lithium treatment of the APPSwDI/NOS2−/− mouse results in decreased tau hyperphosphorylation, increased amyloid deposition, altered neuroinflammation and no change in neurodegeneration or memory.

## Materials and Methods

### Transgenic mice and treatments

The study was approved by the Duke University Institutional Animal Care and Use Committee and conformed to the National Institutes of Health Guide for the Care and Use of Animals in Research. The APPSwDI/NOS2−/− mice (produced by crossing APPSwDI mice [33] with NOS2−/− mice [34]) have been described previously [6]. 19 mice aged four months were assigned to one of two groups, either control diet (N = 8) or lithium diet (N = 11). Both the control diet and lithium diet were obtained from TestDiet (a division of LabDiet Purina-Mills International, Land O' Lakes FL). Lithium diet was formulated at 2 g lithium/kg diet to achieve a dose of 333 mg/kg/day as described previously [35]. Diet was replaced on the cage top weekly and mice were weighed weekly. No statistically significant change in body weight occurred throughout the duration of the study for either treatment group.

### Radial-arm water maze

Mice (12 months old) were tested for memory and learning two days prior to sacrifice using the two-day radial-arm water maze as described in detail previously [36]. Briefly, a six-arm maze is submerged in a pool of water, and a platform is placed at the end of one arm. The mouse receives 15 trials per day for 2 days. The mouse begins each trial in a different arm while the arm containing the platform remains the same. The numbers of errors (incorrect arm entries) are counted over a one-minute period. The errors are averaged over three trials, resulting in 10 blocks for the two-day period (blocks 1–5 are day 1 while blocks 6–10 are day 2). Non-transgenic (N = 7) and NOS2−/− mice (N = 7) aged 12 months were also assessed in the radial-arm water maze to determine transgene-dependent behavior changes.

### Tissue Processing and histology

After injection with a lethal dose of ketamine the mice were perfused intracardially with 25 ml normal saline. Brains were rapidly removed and bisected in the mid-sagittal plane. The left half was immersion fixed in 4% paraformaldehyde, while the right half was snap-frozen in liquid nitrogen and stored at −80°C. The left hemibrain was passed through a series of 10, 20 and 30% sucrose solutions as cryoprotection and 25 µm frozen horizontal sections were collected using a sliding microtome and a freezing stage as described previously [37]. The frozen right hemibrain was pulverized using a mortar and pestle with liquid nitrogen and the brain powder stored at −80°C.

Eight 25 µm sections equally spaced 600 mm apart were selected for free floating immunohistochemistry for Aβ (rabbit polyclonal anti-Aβ N terminal, Invitrogen, Carlsbad, CA. 1∶3,000), neuN (Mouse monoclonal, Millipore, Temecula, CA. 1∶3,000), PHF-tau (AT8, mouse monoclonal for PHF-tau recognizing phosphorylated Ser202 in tau, Thermo Scientific, Rockford, IL. 1∶300), CD45 (Rat monoclonal, Thermo Scientific, Rockford IL. 1∶3,000) and CD11b (Rat monoclonal, AbD Serotec, Raleigh NC. 1∶3,000). The method for free-floating immunohistochemistry has been described previously [6]. Additionally, eight 25 µm sections equally spaced 600 mm apart were selected, mounted on slides and stained in a 0.5% Cresyl violet solution (Sigma-Aldrich, St Louis, MO) for 5 minutes at room temperature. The sections were then differentiated in 70% and 95% ethanol solutions before being coverslipped.

Immunohistochemical product was quantified by assessing percent area occupied by positive stain using the Nikon Elements BR software package (Nikon, Melville NY). Briefly, images were collected on a Nikon Eclipse 90i upright microscope equipped with a Nikon DS-Ri1 digital camera. Specific landmarks on the tissue section were used to ensure the correct regions were examined. Fields of the frontal cortex and hippocampus were localized using 100× magnification followed by image collection at 200× magnification. Representative images were used to establish thresholds using Hue, Saturation and Intensity (HSI) values. The threshold file was saved and then applied to all images from all samples of a given immunostain to yield individual percent area occupied values for each image. Approximately six images of frontal cortex and four images of the hippocampus were assessed for each animal.

### Quantitative real-time RT-PCR

RNA was extracted from approximately 40 mg frozen pulverized tissue using the RNeasy tissue kit (Qiagen, Valencia, CA) according to the manufacturer's instructions. RNA was quantified using the nanodrop spectrophotometer (Thermo Scientific, Rockford IL) and cDNA produced using the cDNA High Capacity kit (Applied Biosystems, Foster City CA) according to the manufacturer's instructions. Real-time PCR was performed using the TaqMan Gene Expression assay kit (Applied Biosystems, Foster City CA) according to the manufacturer's instructions and as previously described [10]. The genes examined are summarized in table 1; all were normalized to 18S rRNA. We determined fold-change compared to non-transgenic mice using the 2 ^(−delta delta Ct)^ method [38].

**Table 1:** Gene expression probes used for the real-time RT-PCR studies.

Gene name	Taqman probe number	RefSeq
ARG1	Mm00475988_m1	NM_007482
IL-1β	Mm00434228_m1	NM_008361
IL-1Ra	Mm00446186_m1	NM_031167
IL-6	Mm00446190_m1	NM_031168
MARCO	Mm00440265_m1	NM_010766
MRC1	Mm00485148_m1	Nm_008625
TGFβ	Mm00441726_m1	NM_011577
TNFα	Mm00443258_m1	NM_013693
TNFαR1	Mm00441875_m1	NM_011609
YM1	Mm00657889_mH	NM_009892

### Western Blot

Approximately 60 mg of the brain powder was homogenized and protein lysates were prepared in M-per lysis buffer (Thermo Scientific, Rockford, IL) containing 1% complete protease/phosphatase inhibitor (Thermo Scientific, Rockford IL). Protein concentrations were assessed using the BCA protein assay kit (Thermo Scientific, Rockford, IL), according to manufacturer's instructions. 15 µg protein from each lysate was run on a denaturing 4–20% SDS-PAGE gel. The gel was transferred onto a PVDF membrane using the iBlot system (Invitrogen, Carlsbad CA), and Western blots were performed for PFH Tau AT8 (Thermo Scientific, Rockford, IL 1∶500) and AT180 (Thermo Scientific, Rockford IL 1∶1000). The blots were stripped using 5× New Blot Nitro Stripping Buffer (Licor, Lincoln NE) and re-probed using the above protocol for with ß-actin as loading control. Semi-quantitative densitometry analysis was performed using the Odyssey Imaging Software (Licor, Lincoln, NE). Individual densitometry values were normalized to the β-actin densitometry value on the same blot.

### ELISA

For ELISA measurement of Aβ we performed a two step protein extraction. 150 mg brain powder was first extracted in 250 µl PBS containing 1% complete protease/phosphatase inhibitor (Thermo Scientific, Rockford IL). The homogenate was centrifuged at 16,000×g at 4°C for 30 minutes. The supernatant was removed and became the “soluble” extract. The resulting pellet was then homogenized in 100 µl 70% formic acid and centrifuged at 16,000×g at 4°C for 30 minutes. The supernatant was removed, neutralized 1∶20 with 1 M Tris-HCl and became the “insoluble” extract. Protein concentration for both the soluble and insoluble extracts was determined using the BCA protein assay according to manufacturer's instructions. We used the Meso-Scale Discovery multiplex ELISA system to measure soluble and insoluble Aβ38, Aβ40 and Aβ42 (MSD, Gaithersburg MD). ELISA kits were run according to the manufacturer's instructions.

### Stereological analysis

Neurons that were positive for cresyl violet were counted in the cornu ammonis 3 (CA3) and the subiculum using the optical fractionator method of stereological counting (West et al., 1991) and the Olympus CAST 1 stereology system (Olympus, Center Valley PA) connected to an upright Olympus microscope. The regions of interests (ROI) were defined using specific landmarks within the hippocampus to maintain consistency. A grid was placed randomly over the region of interest slated for counting. At regularly predetermined positions of the grid, cells were counted within three-dimensional optical disectors. Within each dissector, a 1 µm guard distance from the top and bottom of the section surface was excluded. Section thickness was measured regularly on all collected sections to estimate the mean section thickness for each animal after tissue processing and averaged 14.64 µm±0.29 µm for all sections analyzed. The total number of neurons was calculated using the equation:
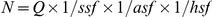
Where N is total neuron number, Q is the number of neurons counted, ssf is section sampling fraction, asf is the area sampling fraction and hsf is the height.

### Statistics

The significance of genotype- and treatment-specific behavioral changes were analyzed by the unpaired Student's *t* test or two-way ANOVA. All immunohistochemical, stereological, ELISA, Western blot and qRT-PCR data were analyzed by one-way ANOVA. The statistical analysis software JMP (Version 9, SAS, Cary NC) was used for all statistical analyses with p<0.05 judged as significant. All graphs were made using Graphpad Prism 4 (GraphPad, San Diego, CA).
